# Friend or foe: inter‐specific interactions and conflicts of interest within the family

**DOI:** 10.1111/een.12259

**Published:** 2015-09-23

**Authors:** ORNELA DE GASPERIN, REBECCA M. KILNER

**Affiliations:** ^1^ Department of ZoologyUniversity of CambridgeCambridgeU.K.

**Keywords:** Animal families, Nicrophorus, social evolution, trophic interactions

## Abstract

1. Interactions between species can vary from mutually beneficial to evolutionarily neutral to antagonistic, even when the same two species are involved. Similarly, social interactions between members of the same species can lie on a spectrum from conflict to cooperation.

2. The aim of the present study was to investigate whether variation in the two types of social behaviour are interconnected. Is the fitness of the various classes of social partner within species (such as parent and offspring, or male and female) differently affected by interactions with a second species? Moreover, can inter‐specific interactions influence the outcome of social interactions within species?

3. The present experiments focus on the interactions between the burying beetle Nicrophorus vespilloides Herbst and the phoretic mite Poecilochirus carabi G. Canestrini & R. Canestrini. The approach was to measure the fitness of burying beetle mothers, fathers, and offspring after reproduction, which took place either in the presence or absence of mites.

4. We found that male, female, and larval burying beetles derive contrasting fitness costs and benefits from their interactions with the mite, despite sharing a common family environment. From the mite's perspective, its relationship with the burying beetle can, therefore, be simultaneously antagonistic, neutral, and possibly even mutualistic, depending on the particular family member involved. We also found that mites can potentially change the outcome of evolutionary conflicts within the family.

5. We conclude that inter‐specific interactions can explain some of the variation in social interactions seen within species. It is further suggested that intra‐specific interactions might contribute to variation in the outcome of interactions between species.

## Introduction

It is well appreciated that inter‐specific interactions vary from the mutually beneficial to the evolutionarily neutral to the antagonistic. However, substantial evidence also shows that the nature of these interactions is not set in stone, but rather varies with the interacting individuals and the ecological context of their interaction (e.g. Douglas, 2010; Thompson, 2013). Furthermore, transitions back and forth from mutualism to parasitism on an ecological timescale are matched by similar transitions on an evolutionary timescale (Boucher, 1985; Machado *et al.,* 2001; Sachs & Simms, 2006; Douglas, 2010). This sort of variation is particularly pronounced within animal families, because the individual members of animal families interact with other species in ways that can be antagonistic (e.g. Kilner & Langmore, 2011), commensal (e.g. Okabe & Makino, 2008), or mutualistic (e.g. Okabe & Makino, 2008). A key outstanding problem is to identify the factors that underpin this variation.

Just as the outcome of inter‐specific interactions can vary from mutualism and parasitism, so the outcome of interactions between species also lies on a spectrum ranging from cooperation to conflict (Davies *et al.,* 2012). This is especially evident in animal families, where parents can cooperate to provision offspring yet remain vulnerable to manipulation by a lazy partner (Lessells, 2012), where siblings are rivals for resources (Mock & Parker, 1997) yet can cooperate to obtain more food (e.g. Schrader *et al.,* 2015) and where offspring reliably advertise their need to provisioning parents (Kilner & Hinde, 2008) yet may seek more than is optimal for parents to supply (e.g. Hinde *et al.,* 2010). The key challenge in this context is to identify the ecological conditions that tip the outcome of these intra‐specific interactions from cooperation to conflict.

Here we address both these problems, by suggesting that they are interconnected. We consider whether ongoing social interactions within a species mean that the interaction with another species can be simultaneously mutualistic, commensal or antagonistic and that the particular outcome depends on where individuals sit in the existing social structure within species. For example, in the context of the animal family, the outcome of an inter‐specific interaction may depend on whether individuals are mothers, fathers or offspring (in other social groups, it may depend on whether individuals are dominant or subordinate, or reproductive or non‐reproductive). We also investigate whether interactions with a second species can tip the balance between cooperation and conflict within a species. Our approach is to analyse interactions between families of burying beetles *Nicrophorus vespilloides* Herbst and the phoretic mite *Poecilochirus carabi* G. Canestrini & R. Canestrini, characterising these relationships by measuring some of the resulting fitness costs and benefits to members of the two species involved.

Burying beetles (*Nicrophorus* spp.) breed upon the body of a small, dead vertebrate, preparing it for reproduction by shaving the carcass, rolling the flesh into a ball, and interring it in a shallow grave. Larvae hatch from eggs laid in the soil nearby and crawl to the carcass where they are fed regurgitated carrion by their two parents, and also self‐feed. Roughly a week after hatching, the larvae disperse away from the scant remains of the carcass to pupate in the soil and the adult burying beetles fly off in search of more carrion. *Poecilochirus carabi* mites are associated with burying beetles because they too require the carcass of a small vertebrate for reproduction (Pukowski, 1933; Scott, 1998). However, unlike the beetle, these mites are incapable of independently locating a fresh corpse and so use the burying beetle as a means of transport between breeding opportunities, riding on a burying beetle to a carcass as sexually immature deutonymphs (an asexual nymphal stage of the mite). *Poecilochirus carabi* mite deutonymphs attach themselves to the beetles, and the beetles transport them between breeding events. In the 36 h after carcass burial, 98% of the mites disembark from the beetles (Wilson & Knollenberg, 1987), moult into adults, and mate. Then, the female mites lay their eggs in the soil around the burial chamber (Wilson & Knollenberg, 1987), and the offspring feed alongside the beetle larvae on the carcass. The next generation of mites disperses mainly on the adult beetles as they disperse away at the end of reproduction (Schwarz & Müller, 1992) to find another carcass (Wilson & Knollenberg, 1987). Thus, the duration of the mite's life cycle closely matches the duration of parental care of the burying beetles (Wilson & Knollenberg, 1987).

In general, the nature of interactions between mites and insects is highly variable. Some mite species are mutualistic, offering ‘cleaning services’ to insects by reducing the pathogenic fungi community in their nests (Biani *et al.,* 2009), or by consuming wastes. Additionally, some mites can promote the transmission and survival of fungi species that have a positive effect on insect fitness (Okabe, 2013). However, mites can reduce the reproductive success of insects by transferring parasitic fungi into the nest (Okabe, 2013), and they can also feed on eggs and larvae, or consume the larvae's food, thus hampering larval development (Cordeiro *et al.,* 2011). Previous analyses of interactions between *Nicrophorus* species and *P. carabi* suggests that the nature of the relationship between the mite and the beetle is similarly variable. *Poecilochirus carabi* mites are potentially beneficial for burying beetles because they have been observed to pierce the eggs of blowflies, which compete with burying beetles to use carrion for reproduction (Springett, 1968). One study has recorded them eating burying beetle eggs too (Beninger, 1993). In addition, it is possible that mites reduce burying beetle fitness through indirect competition simply by consuming the carcass, so taking key food resources from burying beetle larvae (Eggert *et al.,* 1998) and their parents (Boncoraglio & Kilner, 2012), although this possibility has not yet been investigated explicitly. Experiments examining the relationship between mites and the congeneric burying beetle *N. orbicollis* further suggest that mites could provide long‐term fitness benefits for burying beetles but that they have negative effects on burying beetle fitness at very high densities (Wilson & Knollenberg, 1987). Nevertheless, a similar experiment has not been performed with *N. vespilloides* and *P. carabi*.

The overall picture emerging from previous work, therefore, is that the relationship between the mite and the burying beetle depends partly on the presence of rival blowflies and partly on the density of the mite population on each carcass. What remains unclear, however, is whether the nature of the interaction between mites and beetles is different for each member of the family, and whether the mites can heighten the extent of conflict (or cooperation) between members of the same family. To answer these questions, the experiment we describe here measured the fitness consequences of mite–beetle interactions separately for mothers, fathers, and offspring, to test whether they were positive, neutral or negative for each of the different members of the family. By comparing these measures with equivalent measures of fitness taken from families breeding in the absence of mites, we also investigated whether mites influenced evolutionary conflicts of interest or cooperation within the family. For example, we assessed whether mites changed the outcome of sexual conflict over parental investment by examining whether the costs of care were divided differently between the sexes when beetles bred alongside mites compared with breeding in their absence (see Kilner *et al.,* in revision eLife, for similar reasoning). Similarly, we assessed the effect of mites on parent–offspring conflict by examining whether they changed the trade‐off between offspring size and number (see Kilner & Hinde, 2012, for how this trait is affected by parent‐offspring conflict). However, the complex results obtained from previous experiments analysing interactions between burying beetles and mites meant that we were unable to make any explicit directional predictions *a priori*.

## Materials and methods

There are about 14 species of mites belonging to four different families associated with *Nicrophorus* beetles (Wilson & Knollenberg, 1987). We focused on the association between *N. vespilloides* and *P. carabi* (*Arachnia: Acari*) in this study, because this is the most common mite associated with *N. vespilloides* (most mites found on beetles in nature are *P. carabi;* Schwarz *et al.,* 1998) and the most straightforward to culture and manipulate.

### General stock maintenance

#### Laboratory population of burying beetles

All the beetles used in these experiments came from a stock population kept in the Department of Zoology, at the University of Cambridge that was initially founded in 2005 with burying beetles from Madingley woods, Cambridgeshire. Each year, fresh field beetles were brought from the field and bred with the laboratory population between April and September to avoid inbreeding. Before introducing field beetles into the laboratory population, we removed any mites they carried (see below), and cultured them separately, thus keeping the beetle population separate from the *P. carabi* population. All beetles were kept in small plastic containers (12 × 8 × 2 cm^3^) filled with moist soil and fed twice a week with small pieces of minced beef. Adult beetles were breed when they were between 2 and 3 weeks old in plastic breeding boxes (17 × 12 × 6 cm^3^) filled two‐thirds with moist soil with a mouse carcass. The population was maintained in a lab at 20 °C and on a LD 16:8 h.

#### Laboratory population of mites

Freshly caught field beetles were anaesthetised using CO_2_, and all the mites were removed and counted using a brush and tweezers. Mites were kept inside plastic containers (17 × 12 × 6 cm^3^) filled with moist soil. Each mite container also housed a single burying beetle inside, which was fed once a week with minced beef. The containers were kept in cupboards to simulate dark underground conditions, and the mite population was bred once a month. Mites were bred in plastic breeding boxes (17 × 12 × 6 cm^3^) filled two‐thirds with moist soil, alongside burying beetles on a mouse carcass. At the end of the reproductive event, mites dispersing on the adult beetles were collected and re‐introduced to the mite containers. The beetles and larvae that bred alongside the mites were discarded.

### Experiment

All the beetles used in the experiment came from the stock population above, and they developed without any mites. Pairs of sexually mature, virgin beetles were each given a 9–16 g mouse carcass (mean = 12.10 g; SD = 2.35) in a plastic container filled with 2 cm of soil. There were two different treatments. Either pairs were given no mites, or they were given 10 deutonymphs of the *P. carabi* species. This number closely resembles the natural mite load of 4–8 deutonymphs on each wild‐caught *N. vespilloides* at the start of the breeding season (Schwarz & Müller, 1992). Each pair received a ‘pair code’ that had information on the males' and females' pedigree.

According to the precedent established by many previously published studies of *N. vespilloides* from different laboratories, we decided to leave males in the breeding box throughout the experiment (under natural conditions, males leave the brood sometime after hatching but before the larvae disperse, whereas the female stays in the nest until the brood has fully developed De Gasperin Quintero, 2015). Our reasoning was that the presence of mites could change the natural desertion time of the male and thus removing males ourselves midway through brood development, at a fixed, arbitrary time in each treatment, could potentially introduce a confounding effect (see De Gasperin *et al.,* Animal Behaviour, in press). It is extremely unlikely that leaving the males in the breeding boxes confounded our experiments because: (i) although males were unable to leave the breeding box, they were able to leave the carcass and were commonly found at the opposite end of the breeding box after the larvae had hatched; (ii) it introduces no particular bias into our experiments because both treatments experienced this arrangement; (iii) it had no effect on the number of mites carried away from the breeding event by each sex (see ‘Results’).

At the end of the reproductive event (8 days after pairing), we opened all the breeding boxes and removed every larva from the box. We counted and weighed the brood, and anaesthetised both parents using CO_2_ so that we could remove and count the deutonymphs using tweezers and a brush. Note that we also anaesthetised parents from the ‘mite‐free’ treatment in the same way, and also pretended to remove deutonymphs using tweezers and a brush to ensure all beetles from both treatments were treated equally. After the breeding event no parent was kept with any mites, so the individuals in the ‘mite’ treatment only had mites for the duration of the reproductive event (8 days).

To determine the residual fitness of males and females used in the experiment, we kept adults individually in boxes after they had completed their breeding treatments and fed them twice a week with minced beef. We censused the population twice a week to measure the subsequent lifespan and noted the day that adults died and measured their pronotum width (to measure body size) at this point. Lifespan was measured as days after eclosion. In nature, burying beetles are opportunistic breeders, dependent on the essentially random appearance of a carcass to breed. Prolonging survival, therefore, potentially increases fecundity by increasing the likelihood of encountering another corpse. Furthermore, our previous experimental work shows that burying beetles practice reproductive restraint, holding back resources from current reproduction so as to prolong the lifespan, presumably to increase the chance of breeding again (Cotter *et al.,* 2011). For these reasons, lifespan after reproduction is an important component of fitness in the burying beetle (Ward *et al.,* 2009).

We carried out this experiment in five blocks, with replicates of both experimental treatments within each block. We controlled for variation across blocks by including the block as a random effect in our analysis, and included the ‘pair code’ as a random effect nested within the block to control for the genetic pedigree of the beetles. We had a total of 31 successful replicates per treatment.

### Statistical analysis

We analysed the data in the statistical program R (v. 3.0.2; R Development Core Team, 2011). We analysed the success of the beetles with general linear‐mixed effects models. We performed Shapiro tests on all our explanatory variables, and because all of them were normally distributed, we used a normal error distribution in our models (results of the Shapiro tests: average larval mass W = 0.97, *P* = 0.22; brood size W = 0.97, *P* = 0.17; *P* = 0.46, female lifespan W = 0.96, *P* = 0.07; male lifespan W = 0.97, *P* = 0.14). In each model, we included the block as a random effect to control for variation among blocks. We also included a random effect the pair of beetles, and nested this variable within the experimental block. To analyse the success of the brood, we included as response variables the size of the brood, and the average larvae mass (variable obtained by dividing the total brood mass by the brood size). As brood mass and brood size are highly correlated (Pearson's correlation *r* = 0.91; d.f. = 60; *P* < 0.0001), we only included the size of the brood as an explanatory variable in our models. When comparing the average larvae mass, we also included the size of the brood as an explanatory variable as brood size strongly predicts the average larvae mass at dispersal (Schrader *et al.,* 2015). We also calculated effect sizes of the treatment on all our response variables, using Cohen's *d* (Cohen, 1992).

As we were unable to sex the mites that we introduced at the start of the experiment, we could not control the sex ratio of mites we added in our ‘mite present’ treatment (first generation of mites). By chance, in some replicates we may have added 1 female, but in other treatment there may have been as many as 9 (or 10) females present. Consequently, there was considerable variation in the final number of deutonymphs dispersing from the carcass (i.e. the second generation of mites in our experiment) within the ‘mite present treatment’ (see also Nehring & Müller, 2009), which was not accounted for in our analyses by the categorical descriptions of mite presence. Focusing only on the data collected in the ‘mite present’ treatment, we began by investigating whether this variation in the number of second generation mites (variable obtained by adding the total number of mites dispersing on the male and on the female in each family) could account for variation in brood size and in average larvae mass at dispersal (to do this, we assumed that male and female mites have similar effects on burying beetles). Therefore, we performed general linear mixed effects models using brood size as a response variable and included as explanatory variables the final number of second generation mites (log‐transformed), and the mass of the carcass. In a subsequent analysis, we investigated the relationship between the average larval mass at dispersal and the relative density of second generation mites, calculated as log – final number of mites/brood size. We used this measure because the final number of mites and the brood size were correlated and could not be treated as independent explanatory factors (see ‘Results’):

$$\text{mite density}\;by\;\text{brood size}=\frac{\log\;\text{final number of mites}}{\text{brood size}}$$

To analyse the residual fitness of each parent, we used general linear mixed models with treatment (mites present/absent), size of the parents, and mass of the carcass and lifespan as the response variable. We also included the interaction between the size of the parents and the mite treatment. These analyses met the assumptions of the general linear models (see above). We also analysed whether variation in mite number could account for variation in parental lifespan. We performed general linear mixed effects models using lifespan of the male and lifespan of the female as response variables and included as explanatory variables the final number of mites (log‐transformed), and the mass of the carcass.

Finally, we analysed the success of the mites using a general linear mixed model, including as explanatory variables the size of the male and female parent, the carcass mass, and the size of the brood (or the average larval mass, depending on which variable explained more variance) and using the total number of second generation mites dispersing on both parents as the response variable (log‐transformed). To test whether the mites dispersed differently on males and females, we compared the number of mites dispersing on the male and female parent within each pair. For this, we used the number of mites carried by each individual at the end of the experiment as a response variable in a general linear mixed effects model with the lmer function (lme4 package; Bates *et al.,* 2011), and included as explanatory variables the sex of the parent, the size of each individual, and the mass of the carcass. We also included the pair as a random effect.

We obtained *P*‐values for all the models using the ‘anova’ function (‘lmerTest’ package). We reduced every model using the Akaike Information Criterion (AIC; Akaike, 1974) and checked the distribution of the residuals from the final model. Note that this function uses a ‘Satterthwaite’ approximation to calculate the denominator degrees of freedom (Kuznetsova *et al.,* 2013).

## Results

### Effect of mites on the success of the brood

We found no significant effect of the presence of the mites on the size of the brood. However, larvae were on average lighter at dispersal when they developed alongside mites (Table 1). The effect size of the presence of the mites on the success of the brood was small for both variables (Cohen's *d* = 0.31 for brood size, and *d* = 0.24 for the average larval mass). A Levene's test for homogeneity of variance showed that the variance in the ‘mite’ and ‘mite‐free’ treatment was the same for the brood size (*F*_1,60_ = 0.24; *P* = 0.62), and for the average larvae mass (*F*_1,60_ = 0.12; *P* = 0.72).

**Table 1:** Results from the final models for each variable analysed including the mite present and the mite absent treatments

Dependent variable	Factor	d.f. numerator	d.f. denominator	MS	*F* ratio	*P*
Success of the brood						
Brood size	Carcass mass	1	24.48	92.13	2.03	0.16
	Treatment	1	55.46	113.40	2.50	0.11
Average larval mass	Carcass mass	1	50.06	0.001	3.13	0.08
	Treatment	1	53.93	0.003	7.63	0.007*
	Brood size	1	57.97	0.02	61.57	<0.0001*
Residual fitness of the parents						
Male lifespan	Carcass mass	1	9.52	535.25	7.19	0.02*
	Treatment	1	48.01	1166.29	15.68	0.0002*
Female lifespan	Treatment	1	54.85	355.52	6.69	0.01*
	Male size	1	56.93	159.28	3.00	0.08
	Treatment × male size	1	54.58	440	8.28	0.005*

* *P* < 0.05.

**Figure 1:**
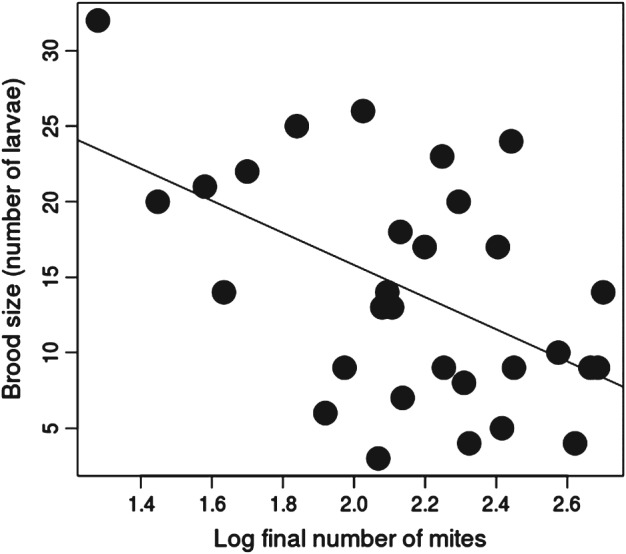
The relationship between the number of second generation mites (log‐transformed) and brood size at dispersal. Each datapoint depicts a different brood. The least squares regression line through the raw data is shown.

**Figure 2:**
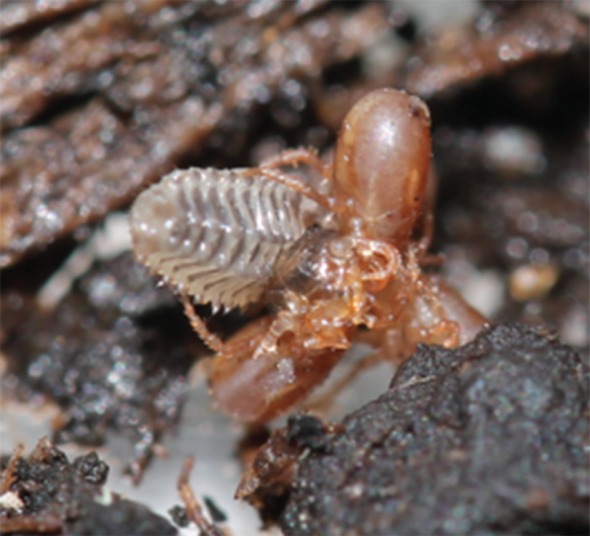
Two mites Poecilochirus carabi attack and feed upon a first instar burying beetle larva Nicrophorus vespilloides. Photo credit. A. Attisano. First instar N. vespilloides larvae are on average 4.87 mm long (SD = 0.85 mm, N = 8).

**Figure 3:**
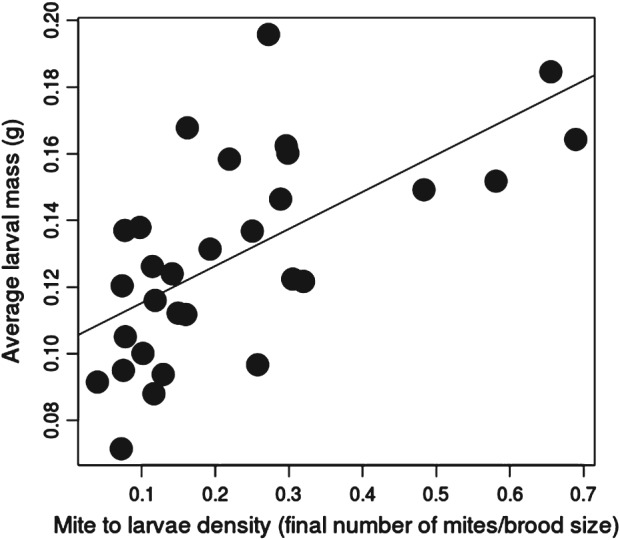
The relationship between the mite density per larva (log number of second generation mites/brood size at dispersal) and average larval mass. Each datapoint depicts a different brood. The least squares regression line through the raw data is shown.

When we analysed the ‘mite present’ treatment alone, we found that the final number of mites (log‐transformed) predicted the final number of larvae. Specifically, there were fewer larvae when there were more mites present at the end of the reproductive event (Table 2; Fig. 1). Do mites attack and kill larvae or does the presence of fewer larvae mean that mites can proliferate? We cannot infer causality in these relationships from the data collected in this experiment. Nevertheless, we have observed mites directly attacking newly eclosed larvae, which is consistent with the former possibility (Fig. 2). Furthermore, the average larval mass was strongly influenced by the relative density of the second generation mites. Larvae were on average heavier when there were fewer siblings and more mites, and lighter when there were many siblings but fewer mites (Table 2; Fig. 3).

**Table 2:** Results from the final models for each variable analysed including only the ‘mite’ treatment

Dependent variable	Factor	d.f. numerator	d.f. denominator	MS	*F* ratio	*P*
Success of the brood						
Brood size	Carcass mass	1	18.73	33.19	1.44	0.24
	Log final number of mites	1	26.85	287.34	12.47	0.001*
Average larval mass	Carcass mass	1	24.34	0.002	4.29	0.03*
	Log final number of mites/brood size	1	26.66	0.01	25.31	<0.0001*
Residual fitness of the parents						
Male lifespan	Carcass mass	1	15.90	207.17	2.31	0.14
	Log final number of mites	1	25.38	27.63	0.30	0.58
Female lifespan	Carcass mass	1	12.27	63.03	1.18	0.29
	Log final number of mites	1	26.16	141.89	2.66	0.11
Success of the mites						
Log final number of mites	Carcass mass	1	17.15	0.26	6.63	0.01*
	Male size	1	24.08	0.13	3.33	0.08
	Average larval mass	1	22.87	0.74	18.64	0.0002*

* *P* < 0.05.

### Effect of the mites on parental lifespan

**Figure 4:**
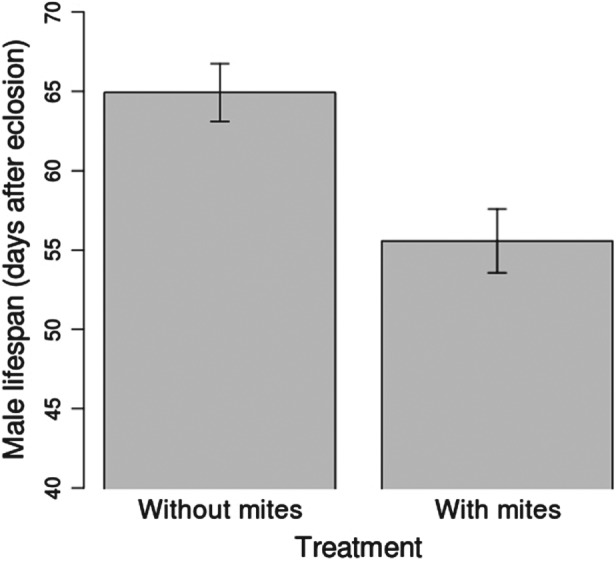
The effect of the presence of mites during reproduction on male lifespan. Means ± SE are shown.

**Figure 5:**
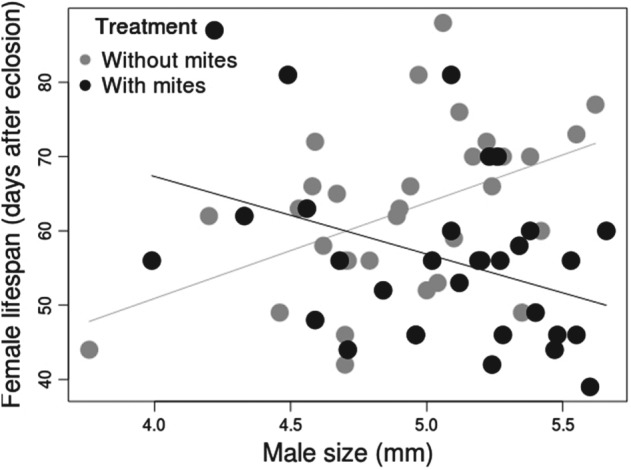
The relationship between the size of the male and the lifespan of the female, when there are mites present during the breeding event (black datapoints, black regression line), and when there are not (grey datapoints, grey regression line). Each datapoint depicts a different brood. The least squares regression lines through the raw data are shown.

The presence of phoretic mites reduced the father's lifespan (Table 1; Fig. 4). The effect size of the mites on the lifespan of the male was large (Cohen's d = 0.87). The presence of the mites also explained variation in the mother's lifespan, but this effect depended on a second variable: the size of her mate (Table 1; Fig. 5). Females had a longer lifespan if they were breeding alongside a large male and there were no mites in the reproductive event. However, when the mites were present, then they had a shorter lifespan if they were breeding alongside a large male. The direct effect of the presence of the mites on the lifespan of the female was medium (d = 0.55). There was no equivalent effect on male lifespan, as neither the interaction between the presence of the mites and the size of the female (P = 0.46), nor the interaction between the presence of the mites and his size (P = 0.88) influenced his lifespan. A Levene's test for homogeneity of variance showed that the variance in lifespan in the ‘mite’ and ‘mite‐free’ treatment was the same for males (F_1,60_ = 0.06; P = 0.80), and females (F_1,60_ = 0.01; P = 0.89). The final number of second generation mites in the reproductive event did not predict either female or male lifespan (Table 2).

### Success of the mites

Within the ‘mite’ treatment, the final number of second generation mites (log‐transformed) was significantly predicted by the average larval mass (Table 2). This variable explained more variation in the final number of mites than the final size of the brood. The mass of the carcass positively predicted the final number of mites as well, and the size of the male had a marginal effect on the final number of mites (Table 2). There were fewer mites in the reproductive event when the male was larger. Mites were just as likely to disperse on males as on females (*P* = 0.29).

## Discussion

We had two aims in this study: to determine (i) whether the outcome of an inter‐specific interaction between mites and burying beetles varies among mothers, fathers, and offspring; and (ii) whether mites change the outcome of social interactions within the family. We have shown that male, female, and larval burying beetles potentially derive contrasting fitness costs and benefits from their interactions with a mite species, *P. carabi,* despite sharing a common family environment. Furthermore, for the offspring, these fitness outcomes change as the density of *P. carabi* mites increases, whereas from the females' perspective the effect of the mites on her lifespan depends on the size of her mate. Therefore, in relation to our first aim, we have shown that the mite's relationship with the burying beetle can be simultaneously antagonistic, neutral, and perhaps even mutualistic, depending on the particular family member involved. Furthermore, in relation to our second aim, we have shown that mites potentially influence the outcome of evolutionary conflicts within the family.

### The effect of mites on maternal, paternal, and offspring fitness

The persistence of the association between mites and burying beetles can seem puzzling in some respects. After summer breeding, adults commonly carry about 40 mites as they fly, which is equivalent to approximately one‐quarter of their body mass (Schwarz & Müller, 1992). When bumblebees, which are similar in size to *N. vespilloides*, fly with pollen loads of equivalent mass to this mite load, they incur substantial energetic costs (Wolf *et al.,* 1989), so it is likely that beetles bear similar costs when transporting mites. Furthermore, mites reduce burying beetle reproductive success, at least at high densities (Wilson & Knollenberg, 1987; Fig. 2). So why has there been no selection on the burying beetle to defend itself more effectively against this seemingly parasitic interaction with the mite? Perhaps the solution to this puzzle lies not only in the fluid nature of the interaction between burying beetles and mites suggested by previous ecological analyses (e.g. Springett, 1968; Wilson & Knollenberg, 1987; Beninger, 1993), but also in the fact that some members of the family gain more from interacting with mites than others. Thus, genes expressed in the mother and some of the offspring may favour continued association with mites, even if genes expressed in fathers do not.

In short, perhaps fluctuating ecological conditions combined with differential benefits offered by mites for different family members, mean that there is unlikely to be directional selection against a continued association between burying beetles and mites, even although this interaction is not beneficial for some members of the family. Thus, in general, our experiments suggest that to understand the variable nature of interspecific interactions, it is important to consider the outcome for different classes of individual within the same species, as defined by their ongoing intra‐specific social interactions. This source of intra‐specific variation might help fuel transitions, back and forth on ecological and evolutionary timescales, from mutualism to parasitism.

### The effect of mites on social interactions within the family

#### Sexual conflict over parental investment between parents

Sexual conflict over parental investment within the family arises over the division of the costs associated with the supply of parental investment (Lessells, 2012). Both parents derive the same benefit from parental investment through offspring fitness, but each would prefer their partner to bear the greater associated cost. Mites can, therefore, potentially influence the outcome of sexual conflict by changing the division of costs associated with the supply of parental investment. This is indeed what we found, measuring the scale of the costs incurred through the effect on parental lifespan (Figs 4 and 5).

Overall, the presence of the mites had a strong, negative effect on male lifespan (Fig. 4). However, from the female's perspective, the effect of the mites on her lifespan depended on the size of her mate (Fig. 5). When paired with larger males, females too suffered a reduction in lifespan when breeding alongside mites. Therefore, when a female is paired with a larger male, the evolutionary interests of the pair are broadly aligned when breeding alongside mites – in the sense that each sex would have a greater lifespan subsequently were the mites not there. However, this is not the case when a female is paired with a smaller male. In this situation, and unlike the male, the female has a relatively long life after reproduction when breeding alongside mites. Therefore, under these conditions, the mites change the outcome of sexual conflict over the division of reproductive costs: females have a longer lifespan after reproduction when mites were present than when they were absent, whereas males have a shorter lifespan. Although there are few equivalent examples in the literature of a second species influencing sexual conflict in a similar way, an experimental study on the guppy, *Poecilia reticulata* W. K. H. Peters showed that the presence of a predator generated sexual conflict between the sexes over courtship patterns (Magurran & Seghers, 1994).

Why did males suffer directly as a result of the presence of the mites, whereas the effect on the lifespan of the female depended on the size of her mate? This effect cannot be explained by a difference in the number of mites dispersing on each sex, as there were no differences in the number of mites that each carried away from the carcass. Furthermore, after the breeding event all the mites were removed from the parents in the ‘mite’ treatment, and beetles were stored without any mites. So it is unlikely that the effect is driven simply by how many mites are on the beetles during the breeding event. Even if mites transferred other parasites or mites to the parents, thus having an indirect effect on the parents, this effect would probably be similar for each sex.

We suggest that the answer instead lies in the way that males and females share resources on the carcass to recoup their costs of reproduction (Boncoraglio & Kilner, 2012). We assume that lifespan after breeding is determined partly by the costs of reproduction (being shorter if greater costs are incurred) and partly by the amount of mouse flesh that males and females consume during reproduction (being longer if more is consumed). Mites can potentially change the way that each of these factors contributes to lifespan. Mites potentially reduce the costs of reproduction because each larva is lighter on average when mites are present (Table 1). This suggests that females, in particular, may sustain lower costs of investment because they contribute more to offspring provisioning (Smiseth & Moore, 2004), and hence to larval mass at dispersal (Lock *et al.,* 2004). Mites may also influence how males and females divide resources on the carcass to recoup their costs of reproduction, somehow allowing the female to take more when her male is small. This can explain why females live longer after breeding alongside mites, but only when their male is small. Why, though, do females live longer after breeding with a larger male in the absence of mites? If we apply the same logic again, either their costs of reproduction are lower, and/or they recoup more of them, when their partner is larger. However, it is not immediately obvious why either possibility should hold true, and detailed behavioural observations are required in future work to understand the processes at work here.

#### Parent–offspring conflict

Mites can potentially influence conflicts of interest within the family in a second way too, through their impact on parent–offspring conflict. When we compared the effect of the two treatments (mite present versus mite absent) on the size of the brood we found no difference between them, but we did find that mites decreased the average larval mass (although the effect size was small). Nevertheless, closer inspection of the data showed that these mean measures concealed considerable variation from brood to brood. Whether mites were present or not, there was considerable individual variation in brood size (and average larval mass), and to a similar extent in each treatment (i.e. mite present or mite absent). For families in both treatments, we showed that some of this variation could be explained by variation in carcass mass, and it was possibly also explained by individual variation in parental quality (which we did not measure). For families in the mite present treatment, the number of mites at dispersal accounted for some of this variation too. When there were relatively few mites present, there were more larvae (Fig. 1), but each larva on average attained a lower mass by dispersal (Fig. 3). With more mites present, there were fewer larvae (Fig. 1) but each on average had attained a greater mass by dispersal (Fig. 3).

There are two possible explanations for this observation. Either high numbers of mites reduce the final size of the brood, or more mites can proliferate when there are fewer larvae present. We suggest that the first explanation is more likely because we have observed mites directly cannibalising recently eclosed larvae (Fig. 2). Therefore we contend that at high densities, mites reduce the size of the brood thereby allowing each individual surviving larva to obtain a greater share of resources and thus to attain a greater mass by the time of dispersal (Schrader *et al.,* 2015).

What does all this mean for parent–offspring conflict? Long‐term field studies on diverse taxa consistently show that the optimal trade‐off between offspring size and number favoured by natural selection is different for parents and their individual young (reviewed in Kilner & Hinde, 2012). Whereas parents maximise their number of descendants by producing more and smaller offspring, individual offspring leave more descendants if they are from a smaller brood and so attain a greater size at birth or independence (reviewed in Kilner & Hinde, 2012). If this pattern is true for burying beetles as well, then our data suggest that mites can potentially influence the trade‐off between offspring size and number in burying beetles, perhaps tipping the balance towards the parental optimum when mite numbers are low, but towards the optimum for (surviving) offspring at higher mite densities (Figs 1 and 3).

More generally, our experiments indicate that the outcome of evolutionary conflicts of interest within the burying beetle family can be influenced by inter‐specific interactions that are imposed on the family to some degree. Whereas it is commonly supposed, or predicted, by theoretical analyses of conflicts of interest within the family (cf Godfray, 1995; Lessells, 2012) that at least some members of the family have full control over the provision of parental investment, and that conflicts will be resolved in an evolutionarily stable way, our experiments suggest that neither is likely to be true in the real world. Interactions with other species, which vary in their intensity from generation to generation, can potentially change the outcome of intra‐familial conflicts and make an evolutionarily stable outcome impossible.

In conclusion, we have shown that the evolutionary outcomes of inter‐ and intra‐specific interactions are closely connected. Social interactions within species create distinct social categories, and the outcome of interactions with a second species can vary simultaneously across these different social categories. Transitions from mutualism to parasitism (and vice versa) that are seen on ecological and evolutionary timescales might be influenced by this source of intra‐specific variation, and it would be interesting to explore this possibility in more detail in future work. Furthermore, we have shown the outcome of social interactions within species can be influenced by interactions with a second species. Perhaps future theoretical analyses of social evolution should more explicitly acknowledge the variable ecological context of whichever social interaction is being modelled and consider the possibility that behavioural strategies might never attain an evolutionary equilibrium in a real and ever‐changing world.
